# Treatment of distal clavicle fracture of Neer type II with locking plate in combination with titanium cable under the guide

**DOI:** 10.1038/s41598-021-84601-2

**Published:** 2021-03-02

**Authors:** Jun Wang, Jie Guan, Minbo Liu, Yongfeng Cui, Yuhang Zhang

**Affiliations:** Department of Orthopedic, Xiaoshan 1st People’s Hospital, No. 199 Shixin South Road, Hangzhou, 311200 Zhejiang China

**Keywords:** Biological techniques, Biotechnology

## Abstract

To observe and compare the curative effect of a locking plate plus titanium cable under the Guide device and clavicular hook plate in the treatment of Neer type II distal clavicle fractures. A prospective cohort study was conducted to analyse the clinical data of 36 patients with distal clavicle fractures from January 2016 to January 2019. The results were analysed. According to the random number method, the patients were divided into two groups: the titanium cable group (fixed with a titanium cable in combination with a locking plate) and hook plate group (fixed with a clavicular hook plate only). Under the guidance of a special device (for which a patent was obtained), in the titanium cable group, the coracoclavicular ligament was fixed with tension reduction, and then the distal clavicular fracture was fixed with a locking plate. In the hook plate group, the distal clavicle fracture was fixed with a hook plate. The incision length, operation time, bleeding volume and VAS score before, 1 week after and 1 year after the operation were compared between the two groups. The effect of the operation was evaluated by the Constant-Murley score before and 1 year after the operation. X-ray films were taken 2 days, 3 months, half a year and 1 year after the operation to observe the reduction and healing of fractures. At the same time, complications were recorded. The amount of bleeding was the same in the two groups. The operation time in the hook plate group was relatively short, and the difference was statistically significant (P < 0.05). The VAS score in the titanium cable group was significantly lower than that in the hook plate group one year after the operation. The Constant-Murley score in the titanium cable group and hook plate group was significantly higher 1 year after the operation. The number of postoperative complications in the titanium cable group was significantly lower than that in the hook plate group. The treatment of Neer type II distal clavicle fractures with a titanium cable plus a locking plate has a good curative effect, few complications and good postoperative recovery and thus is worth popularizing.

## Introduction

Clavicle fracture is a common fracture that accounts for 2.6–5.0% of all fractures in adults and 35–40% of shoulder injuries, and distal clavicle fractures account for approximately 20% of all fractures of the clavicle^1^. The Neer classification is often used for distal clavicle fractures; the type II fracture with rupture of the conoid ligament is a type of distal clavicle fracture in which the proximal fragment of the clavicle is significantly displaced superoposteriorly to the acromioclavicular joint, with a high likelihood of instability^2^. Rokito et al.^3^ discovered that type II fractures were more prone to non-union (about 22–44%) for conservatively managed fractures, particularly when they were displaced because of CC ligament injury and counterpull of the trapezius on the proximal fragment. Patients experiencing non-union will experience activity limitations, shoulder pain, and so on, which has led to early surgical management of these fractures^4^. A variety of surgical techniques have been used to address unstable distal clavicular fractures, including locking compression plate fixation of the distal clavicle, hook plating, anchor suture fixation, and arthroscopic reconstruction of the conoid ligament^5–8^. However, no consensus has been reached regarding the optimal fixation method, as they are associated with various complications. Locking plate fixation and poor fixation remain challenging, and no definitive solution has been identified^9^. The hook plate is often associated with shoulder pain, non-union, and subacromial impingement or the need for hook plate removal^10–12^. Anchor suture fixation and arthroscopic reconstruction of the conoid ligament are associated with bone tunnel enlargement, clavicle or coronoid fractures, and reduction loss^8,13^. Ideal fixation of Neer type II distal clavicular fractures should provide enough stability to improve the union of fractures and the substantial deforming force on the fracture while avoiding implant irritation and the need for implant removal. In this study, we report our surgical procedure for using CC ligament reconstruction with a titanium cable under the Guide and a locking plate to fix the clavicle. The purpose of our study was to investigate which of these two techniques (titanium cable in combination with plate and hook plate) are more applicable for treating Neer type II distal clavicle fractures.

## Materials and methods

This is a prospective study performed in our hospital from January 2016 to January 2019. This study was approved by the Ethics Committee of the Xiaoshan 1st People’s Hospital of China. The study was performed in accordance with relevant guidelines and regulations. Written informed consent was obtained from all patients before surgery. A consecutive series of thirty-six patients with Neer type II distal clavicle fractures were surgically treated in our trauma centre.

The inclusion criteria were as follows: (1) unilateral acute closed fractures, (2) Neer type II distal clavicle fractures with displacement, (3) internal fixation with a locking plate in combination with a titanium or hook plate, (4) normal shoulder function before the injury, and (5) age greater than 18 years.

The exclusion criteria were as follows: (1) chronic injury, (2) open fractures, (3) shoulder joint arthritis, and (4) other types of clavicle fractures such as those of the coracoid process or acromion.

Thirty-six patients who voluntarily agreed to the study and signed the consent form were randomly divided into two groups for surgical treatment with lock plate fixation in combination with a titanium cable (titanium cable group) and a clavicular hook plate (hook plate group).

Titanium cable (TC) group: an average age of 39.7 years (range from 22 to 68 years); 7 females; 11 males; injuries were classified as Neer type II after a median follow-up of 13.5 months (range from 12 to 18 months).

Hook plate (HP) group: a median age of 39.4 years (range 21–70 years); 8 females; 10 males; injuries were classified as Neer type II after a median follow-up of 13.7 months (range 12–18 months).

### Surgical procedure

#### Titanium cable (TC) group

**Figure 1:**
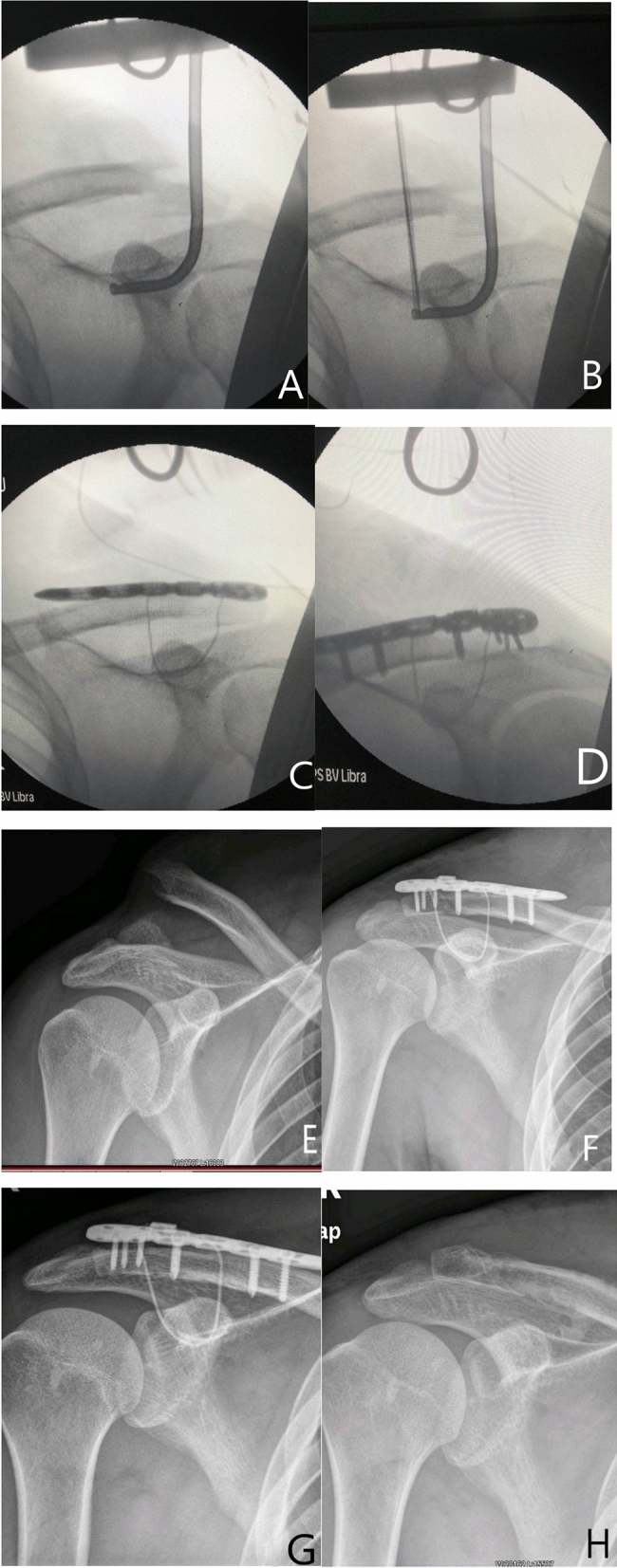
(**A**,**B**) The guide was inserted through the tunnel. (**C**,**D**) A titanium cable was inserted and the clavicle distal locking plate was fix. (**E**–**H**) A 27 years old man with right distal clavicle fracture. The distal fragment more than 2.5 cm, a titanium cable was inserted and the clavicle distal locking plate was fix. (**E**) Preoperative X-ray of the shoulder; (**F**) postoperative X-ray, (**G**) 3 months after operate and fracture healing, (**H**) the final follow-up 12 months after surgery and the implant removed.

The operation was performed under general anaesthesia. The patient was placed in a beach chair position, his shoulders were padded with a 6-cm-thick cushion, and the head was biased to the healthy side to ensure that the clavicle had sufficient passage and that the patient's arm remained adducted. The surgeon faced the operative shoulder and marked the approximate contour of the distal end of the clavicle and the shoulder. A fluoroscopy unit with a C-arm (to visualize the entirety of the clavicle from anteroposterior and apical oblique angles) was positioned on the contralateral side. An obese person was positioned through a C-arm. A skin incision of approximately 4–5 cm was made at the inner edge of the shoulder peak from the lower edge of the lateral edge of the clavicle. The skin and subcutaneous tissue were incised, and the dislocated acromioclavicular joint was exposed. The free torn tissue of the joint was cleaned, and the vascular clamp was inserted along the lateral edge of the base of the coracoid process, which was confirmed under C-arm guidance. The tunnel was created, and the outer end of the clavicle was marked. The Guide was inserted into the lower edge of the coracoid process along the tunnel at the clavicular mark, and the Guide was confirmed to be closer to the lower edge of the base of the coracoid process under the C-arm. The side hole of the Guide slightly exceeded the inner edge of the coracoid process (Fig. 1A). The positioning pin (1 mm K-wire) was inserted from the positioning hole of the Guide 4–4.5 cm posteromedial to the tip of the distal clavicle. After the 3 mm hollow drill was reamed, the 2.5 mm hollow positioning pin was inserted into the side hole of the Guide. After C-arm fluoroscopy, the positioning pin and the guide were integrated (Fig. 1B). The titanium cable was inserted from the hollowed positioning pin through the Guard. The Guard and hollow positioning pin were removed, and a 2.5 mm hole portal was drilled 2 cm anterolateral from the distal end of the clavicle. The distal titanium cable was inserted into the clavicle. The distal clavicle locking plate or T-shaped metacarpal lock-plate was placed according to the size of the distal fragment (Fig. 1C). After fracture reduction, K-wires were temporarily used for fixation, 5–7 screws were implemented, and the titanium cable was locked (Fig. 1D).

#### Hook plate (HP) group

The operation was performed under general anaesthesia. The patient was placed in a beach chair position, his shoulders were padded with a 6-cm-thick cushion, and a skin incision of approximately 8 cm was made at the inner lateral edge of the clavicle. The clavicle fracture line was exposed, and the fracture and AC joint were exposed. After fracture reduction, a K-wire was temporarily used for fixation, and the clavicular hook plate was positioned with the hook dorsally under the acromion and fixed to the clavicle with screws. The K-wire was removed. X-ray was performed. The deltoid-trapezoid fascia was closed with resorbable sutures, and the wound was closed in layers.

### Postoperative management

Following surgery, sling immobilization was used for 1 month. Passive external rotation was encouraged. After 1.5 months, active movement was encouraged if no implant relaxation occurred. Strengthening was initiated at 3 months postoperatively. Return to sports or work was allowed for at least 6 months according to the imaging examination results.

All patients who survived were clinically and radiographically followed for a mean of 13.5 (ranging from 12 to 18) months. The follow-up evaluations were performed in a standardized fashion by two examiners who were not the operating surgeons. Clinical assessment of the operation time, intraoperative haemorrhage, incision size, postoperative complications including infection, malunion, and nonunion, VAS pain scores and Constant-Murley shoulder function scores were observed and recorded. X-ray films were taken 2 days, 3 months, 6 months and 1 year after the operation to observe the reduction and maintenance of the fractures.

### Statistical analysis

Distributions of data were analysed by SPSS 22 (American SPSS company) statistical software, including the measurement data (mean ± SD) of the operation time, intraoperative bleeding volume, incision size, VAS score, and Constant-Murley score^14^, and the Mann–Whitney U test (for VAS score) and the t-test of two independent samples (for others) was used; the measurement data were expressed by the number of cases (percentage), and the X^2^ test was used. P < 0.05 was considered statistically significant.

### Ethics approval and consent to participate

Approved by ethics committee of Xiaoshan 1st People’s Hospital.

### Consent for publication

Since the patients were already deceased when this cases were written,patient’s next of kin signed a written consent from indicating who aware of this case report and the possibility of it being published.

## Results

All 36 patients were followed for 13.5 months (ranging from 12 to 18). No incision infections were found in any patients. There were no nerve or blood vessel injuries.

### Comparison of preoperative data

The 36 patients were randomly divided into two groups. There was no significant difference in age, sex, side, cause of injury, time from trauma to operation, follow-up time, and BMI between the two groups (P > 0.05) (Table 1).

**Table 1:** Comparison of preoperative general data.

Patient demographics	TC	HP
n	18	18
Sex, female/male	6/12	7/11
Age (years)	39.7 ± 15.6 (22–68)	39.4 ± 18.5 (21–70)
Side of injury, left/right	5/13	7/11
Dominant/non-dominant hand	6/12	6/12
Fall down	10/18	9/18
Sport injury	3/18	3/18
Traffic injury	5/18	6/18
Follow-up time (month)	13.5 ± 4.5	13.7 ± 3.8
Time to operation (day)	4.2 ± 1.4	4.0 ± 1.8
Body mass index (BMI)	23.5 ± 8.8	21.9 ± 6.5

### Comparison of intraoperative indexes

There was no significant difference in blood loss,incision length, Fracture healing time between the titanium cable group and the clavicular hook group. There was a significant difference between the two groups in the operation time, with 65 ± 12 min in the titanium cable group and 41 ± 13 min in the clavicular hook group (t = 4.90, P < 0.0001) (Table 2).

**Table 2:** Comparison of intraoperative and postoperative data.

Patient demographics	TC	HP	t/X^2^	P
n	18	18		
Incision length (cm)	8.2 ± 1.5	8.3 ± 1.2	0.2	0.89
Operative time (min)	65 ± 12	41 ± 13	4.90	0.000
Blood loss (ml)	70.2 ± 10.5	69.5 ± 16.3	0.15	0.68
Fracture healing time (week)	10.5 ± 6.5	11.2 ± 4.5	0.19	0.56

### Comparison of VAS

In the titanium cable group, the VAS pain score was 6.1 ± 1.3 points (4–8 points) before the operation, 2.2 ± 0.9 points (1–4 points) one week after the operation, and 0.2 ± 0.4 points (0–1 points) 12 months after the operation, and the difference was statistically significant (median = − 6, P < 0.0001).

In the hook plate group, the VAS pain score decreased from 6.3 ± 1.5 before the operation to 3.4 ± 0.8 (2–5) one week after the operation and 0.9 ± 1.2 (0–3) 12 months after the operation, and the difference was statistically significant (median = − 5, P < 0.0001). Preoperatively, no statistically significant difference was observed between the two groups (P > 0.9999). While, there was a significant difference in the VAS score between the two groups one week after operation and one year after operation (P = 0.0384, P = 0.0131) (Table 3).

**Table 3:** Comparison of VAS score.

Patient demographics	TC	HP	Mann–Whitney U	P
n	18	18		
VAS (preoperative)	6.1 ± 1.3^a^	6.3 ± 1.5^b^	162	0.9999
VAS (1 week after operation)	2.2 ± 0.9	3.4 ± 0.8	99	0.0384
VAS (1 year after operation)	0.2 ± 0.4^a^	0.9 ± 1.2^b^	89	0.0131

^a^Compared with VAS (pre-operate) and VAS (1 year after operate) of TC group, Wilcoxon test, median = − 6, P < 0.0001.

^b^Compared with VAS (pre-operate) and VAS (1 year after operate) of HP group, Wilcoxon test, median = − 5, P < 0.0001.

### Comparison of constant-Murley score

In the titanium cable group, The constant-Murley shoulder function score was 32.6 ± 4.1 (28–38) before the operation and 94.6 ± 6.1 (91–99) 12 months after the operation, and the difference was statistically significant (t = 49.8, P < 0.001).

In the hook plate group, The constant-Murley shoulder function score was 31.1 ± 3.1 (28–36) before the operation and 90.2 ± 3.9 (87–98) 12 months after the operation, and the difference was statistically significant (t = 65.3, P < 0.001).

There was also a significant difference in the postoperative Constant-Murley score between the two groups (t = 4.6, P < 0.001) (Table 4).

**Table 4:** Comparison of constant‑Murley score (CMS).

Patient demographics	TC	HP	t/X^2^	P
n	18	18		
CMS (pre-operate)	32.6 ± 4.1^a^	31.1 ± 3.1^b^	1.1	0.31
CMS (1 year after operation)	94.6 ± 6.1^a^	90.2 ± 3.9^b^	4.6	0.000

^a^Compared with CMS (pre-operate) and CMS (1 year after operate) of TC group, t = − 49.8, P = 0.000.

^b^Compared with CMS (pre-operate) and CMS (1 year after operate) of HP group, t = − 65.3, P = 0.000.

### Comparison of postoperative imaging

Fracture union occurred in all patients at an average of 4.9 months after surgery. In the hook plate group, there were 5 cases of distal clavicular bone resorption and subacromial bone resorption. In the titanium cable group, all cases of fracture union occurred without implant loosening or implant breakage. At the final follow-up, all implants were removed in the hook plate group, and 14 implants were removed in the titanium table group; no refracture occurred after implant removal.

### Comparison of postoperative complications

Four patients complained about postoperative pain around the shoulder in the hook plate group, and disrupted sleep using oral nonsteroidal anti-inflammatory and analgesic drugs was alleviated and disappeared after implant removal. There were no significant complications in the titanium cable group after the operation.

## Discussion

**Figure 2:**
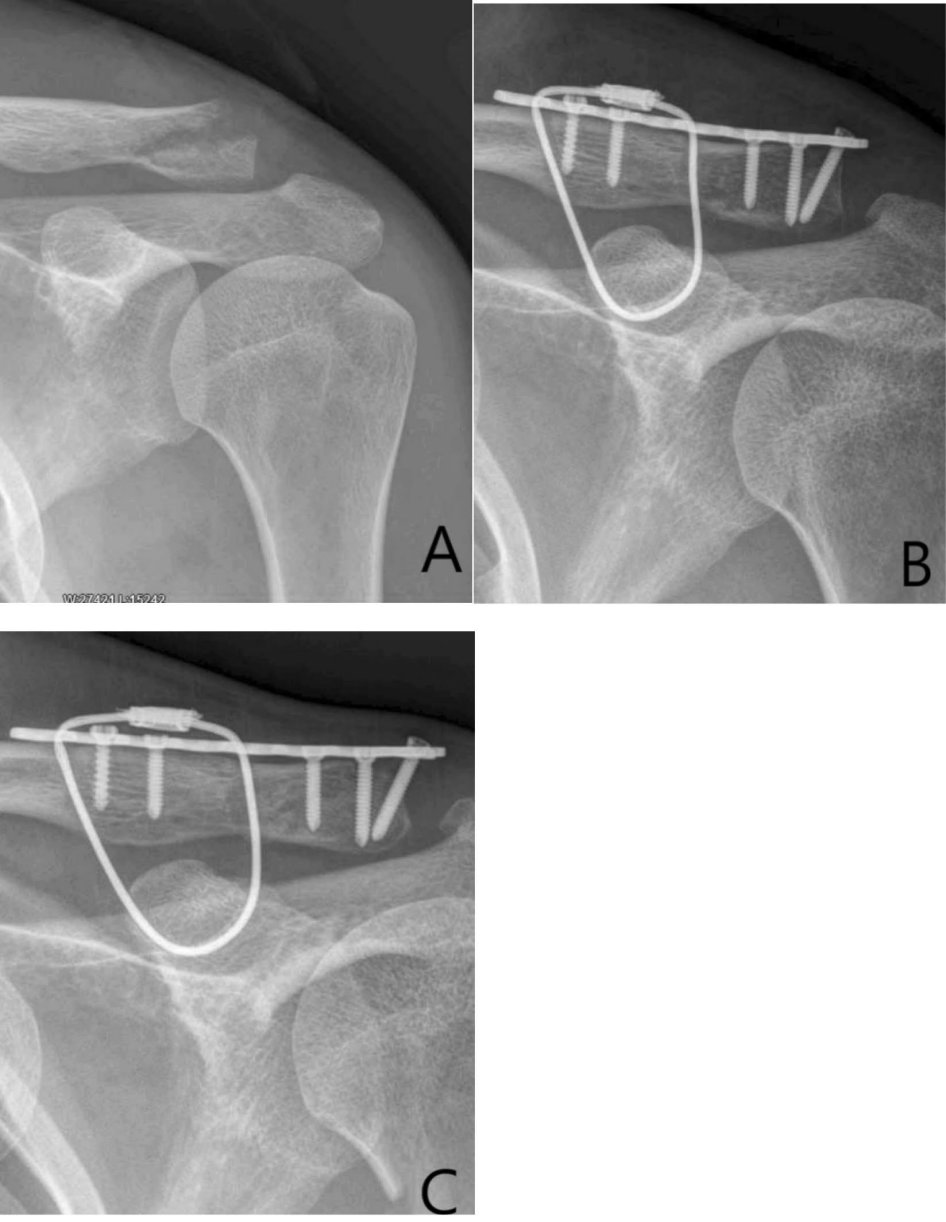
(**A**–**C**) A 36 years old woman with left distal clavicle fracture. The distal fragment less than 2.5 cm, a titanium cable was inserted and the metacarpal locking plate was fix. (**A**) Preoperative X-ray of the shoulder; (**B**) postoperative X-ray, (**C**) 3 months after surgery show fracture union.

The injury mechanism of a distal clavicle fracture is usually a direct fall onto the shoulder with the arm adducted or direct impact to the shoulder, which is consistent with the injury mechanism of acromioclavicular joint dislocation. The typical displacement features of Neer type II fractures are that the fracture line is located on the medial or lateral side of the trapezoid ligament, the lateral fragment remains in place, and the proximal fragment is displaced due to injury to the conoid ligament and/or trapezoid ligament. We divided the length of the distal fragment into two types: type A was a length more than 2.5 cm, representing a typical case for type A (Fig. 1E–H); type B was a length less than 2.5 cm, representing a typical case for type B (Fig. 2A–C). There are three reasons for this division: first, 2.5 cm is the footprint of the trapezoid ligament on the clavicle to determine whether the distal fracture is attached to the trapezoid ligament, and the distal end of the titanium cable is the same as the locking footprint of the trapezoid ligament; second, when the anatomical locking plate of the distal clavicle is less than 2.5 cm, the distal screw cannot produce effective fixation (especially in osteoporosis patients), and plate placement is difficult, which will affect the acromioclavicular joint. In the TC group, the distal clavicle locking plate should be placed for type A fractures; for type B fractures, the author uses T-shaped metacarpal locking plate fixation because the distal end of the fracture can be fixed with three screws without interference of the acromioclavicular joint. Third, Raymond^15^ found that the area of the distal clavicle containing the highest BMD and greatest cortical thickness was at least 2 cm from the lateral edge of the clavicle. We think that the coracoclavicular ligament should be reconstructed or reduced regardless of the type of plate placed because Neer type II fractures are accompanied by coracoclavicular ligament rupture. If the coracoclavicular ligament is not reconstructed or reduced, fracture non-union will occur due to the loosening of screws or the failure of internal fixation^9^. We used different locking plates (clavicle locking plate or metacarpal locking plate) to fix type A or B unstable distal clavicle fractures. In addition, titanium cables were implant under the Guide (patent applied) to reconstruct the coracoclavicular ligament without exposing the coracoid process and without damaging nerves and blood vessels around the coracoid process. Fixation of the clavicle with the double-stranded titanium cable in the area of the coracoclavicular footprint of the coracoclavicular ligament can provide strong stability between the clavicle and the coracoid process; the stress force on the fracture is markedly reduced, and the fracture becomes stable. Many experts have reported that when using only the suture anchor or endobutton to fix the clavicle, reconstruction of the coracoclavicular ligament can only solve the vertical stress from biomechanics of fracture fragment, and horizontal stress still exists. All the cases reported in the literature are suitable for distal fragments within 2.5 cm^16,17^, and the proximal fragment is a short oblique fracture. If the distal fragment is more than 2.5 cm and there are transverse fractures or long oblique fractures, this method would induce many complications, such as nonunion or fracture displacement, and coracoid fixation has specific complications, such as coracoid fractures and neurovascular injuries around the coracoid. In recent years, many authors have used arthroscopic reconstruction of the coracoclavicular ligament to treat distal clavicular fractures, which has the advantage of less damage, but there are still complications such as loss of reduction and coracoid process or clavicular fractures^18,19^. Tae KangLim^13^ et al. used arthroscopic reconstruction of the coracoclavicular ligament to treat 18 cases of acromioclavicular joint dislocation and distal clavicular fracture. Damage to the coracoid process tunnel during the operation was found in 7 patients who had to undergo open surgery. Nine patients had complications after the operation, including 4 with tunnel expansion, 4 with reduction loss, 1 with a coracoid process fracture, and 1 with distal clavicle fracture reduction loss who had to undergo clavicular hook plate repair. Shin Kim^20^ also reported 18 cases of coracoclavicular ligament reconstruction under arthroscopy and 8 cases of reduction loss, fracture and other complications. The outcome of minimally invasive coracoclavicular fixation with a double button in distal clavicular fractures by Süleyman Semih Dedeoğlu^21^ was highly successful; however, 2 patients were not able to return to work because of shoulder pain. Our method was suitable for all distal clavicular fracture patterns, and it offers many advantages, including stable fixation, fast recovery, minimal damage and less than 3 mm of drilling on the clavicle without exposing the coracoid process; there were no fractures of the coracoid process or clavicle. The reduction of the coracoclavicular ligament with titanium cables and fracture fixation with locking plates play a double stabilizing role, so there were few complications in the titanium cable group, no internal fixation loosening after the operation, and no fracture nonunion or refracture; additionally, the locking plate reduced the potential need for hardware removal. Two patients in the TC group were not willing to undergo internal fixation removal, and they had no pain or dysfunction, which also confirmed the advantages of this method.

Another type of operation is fixation with a clavicular hook plate, which is a reliable technique for distal clavicle fractures. In addition, the subacromial hook offers distal leverage that provides reduction of the displaced proximal fragment and may be suitable for all distal clavicular fractures. Because it is simple, safe, and achieves a comparable functional outcome and high union rate, this method is used abroad in the treatment of distal clavicle fractures^22,23^. The treatment of distal clavicular fractures with a clavicular hook plate can usually result in good imaging results and shoulder joint function. However, because of its non-compliance with biomechanics, the size of the acromion and the height of the distal clavicle in each patient are different, which leads to excessive lifting of the acromion and reduces lifting stress of the hook tip. The incidence of postoperative complications is high, mainly including subacromial impingement signs, subacromial bone fusion, shoulder pain, a restricted shoulder abduction range of motion, clavicular hook prolapse, clavicle and acromion fractures, etc.^24,25^. It has been reported that the incidence of shoulder pain after clavicular hook plate fixation is higher than that after distal clavicular locking plate fixation^12^. In the HP group, 5 patients had distal clavicular bone resorption and subacromial bone resorption, and 4 patients had shoulder pain and restricted shoulder abduction. All patients required implant removal.

The shortcoming of this study is as follows: due to the small number of patients in this group, the follow-up time was not long enough, so the long-term effect is still unknown.

## Data Availability

This is a case series, to protect privacy and respect confidentiality; none of the raw data has been made available in any public repository. The original reports, imaging studies and outpatient clinic records are retained as per normal procedure within the medical records of our institution.

## Abbreviations

HP: Hook plate
TC: Itanium cable
VAS: Visual analogue scale
BMI: Body mass index
